# Synthesis, structure and computational study of 1-acetyl-3-(3,5-di­chloro­phen­yl)thio­urea

**DOI:** 10.1107/S2056989026010121

**Published:** 2026-10-02

**Authors:** Sharatha Kumar, Tuncer Hökelek

**Affiliations:** a Department of Chemistry Yenepoya Institute of Arts Science Commerce and Management Mangaluru Yenepoya University (Deemed to be university) 575013 Karnataka India; b Hacettepe University, Department of Physics, 06800 Beytepe-Ankara, Türkiye; Vienna University of Technology, Austria

**Keywords:** crystal structure, acet­yl, thio­urea, 3,5-di­chloro­phen­yl, disorder

## Abstract

The title compound, C_9_H_8_Cl_2_N_2_OS, consists of di­chloro­phenyl and acetyl moieties attached to the N atoms of the bridging thio­urea group. The dihedral angle between the acetyl group and the thio­urea group is 5.6 (6)°, and they are oriented with respect to the phenyl ring at dihedral angles of 70.3 (4) and 69.1 (1)°, respectively. The mol­ecular conformation features an intra­molecular N—H⋯O hydrogen bond with an *S*(6) ring motif. In the crystal, C—H⋯Cl and double-acceptor N—H⋯S and C—H⋯S hydrogen bonds link the mol­ecules, enclosing *R*^1^_2_(6), *R*^2^_2_(8) and *R*^2^_2_(20) ring motifs, into infinite chains running parallel to the *b* axis. Neither π⋯π nor C—H⋯π(ring) inter­actions are observed. Hirshfeld surface analysis revealed that the most important contributions for crystal packing are H⋯H (28.6%), H⋯Cl/Cl⋯H (22.9%), H⋯S/S⋯H (10.4%), H⋯O/O⋯H (9.6%) and H⋯C/C⋯H (9.0%) inter­actions. The volume of the crystal voids and the percentage of free space were calculated to be 62.7 Å^3^ and 10.9%. N—H⋯S, C—H⋯S and C—H⋯Cl hydrogen-bonding energies of −10.0, −3.9 and −7.7 kJ mol^−1^, respectively, were computed. Evaluations of the electrostatic, dispersion and total energy frameworks indicate that the crystal consolidation is dominated by dispersion energy contributions.

## Abstract 2

In the crystal, C—H⋯Cl and double-acceptor N—H⋯S and C—H⋯S hydrogen bonds link the mol­ecules into infinite chains parallel to the the *b* axis.

## 1. Chemical context

Acyl­thio­urea derivatives exhibit a broad range of biological activities, including anti­cancer, anti­microbial, anti-inflammatory, anti­consulvant, anti­diabetic and urease-inhibitory effects (Bielenica *et al.*, 2016 ▸; Ashgar *et al.*, 2018 ▸; Khan *et al.*, 2014 ▸, Canatar *et al.*, 2023 ▸). In particular, thio­urea-based compounds have been investigated as anti­cancer agents because the thio­urea pharmacophore provides hydrogen-bonding sites that can facilitate inter­actions with biological targets, while variations of the N-substituents allow modulation of their mol­ecular and electronic properties (Mishra & Batra, 2013 ▸; Nammalwar *et al.*, 2013 ▸). N-substituted acyl­thio­ureas are also useful synthetic inter­mediates for the preparation of heterocyclic compounds and have been investigated as anion receptors, organocatalysts and corrosion inhibitors (Aly *et al.*, 2016 ▸; Bregovic & Basaric, 2015 ▸; Guang *et al.*, 2015 ▸; Al-Abbasi *et al.*, 2023 ▸). Furthermore, the presence of sulfur and carbonyl oxygen donor atoms gives the compounds versatile metal-coordination properties (Saeed *et al.*, 2013 ▸, 2017 ▸).

The structural versatility of acyl­thio­ureas is reflected in their mol­ecular conformations and supra­molecular assemblies in the solid state. Crystallographic studies have shown that acyl­thio­urea derivatives can adopt conformations consolidated by intra­molecular N—H⋯S and N—H⋯O hydrogen bonds, while inter­molecular hydrogen bonds contribute significantly to the formation of extended supra­molecular networks (Gowda *et al.*, 2012*b* ▸; Kumar *et al.*, 2012*a* ▸; Yusof *et al.*, 2012 ▸). The mol­ecular conformation and hydrogen-bonding patterns are influenced by the nature of substituents and by the conjugated acyl­thio­urea frameworks (Saeed *et al.*, 2014 ▸).

As part of our studies in this area, we now describe the synthesis and structure of the title compound, C_9_H_8_Cl_2_N_2_OS, (**I**).
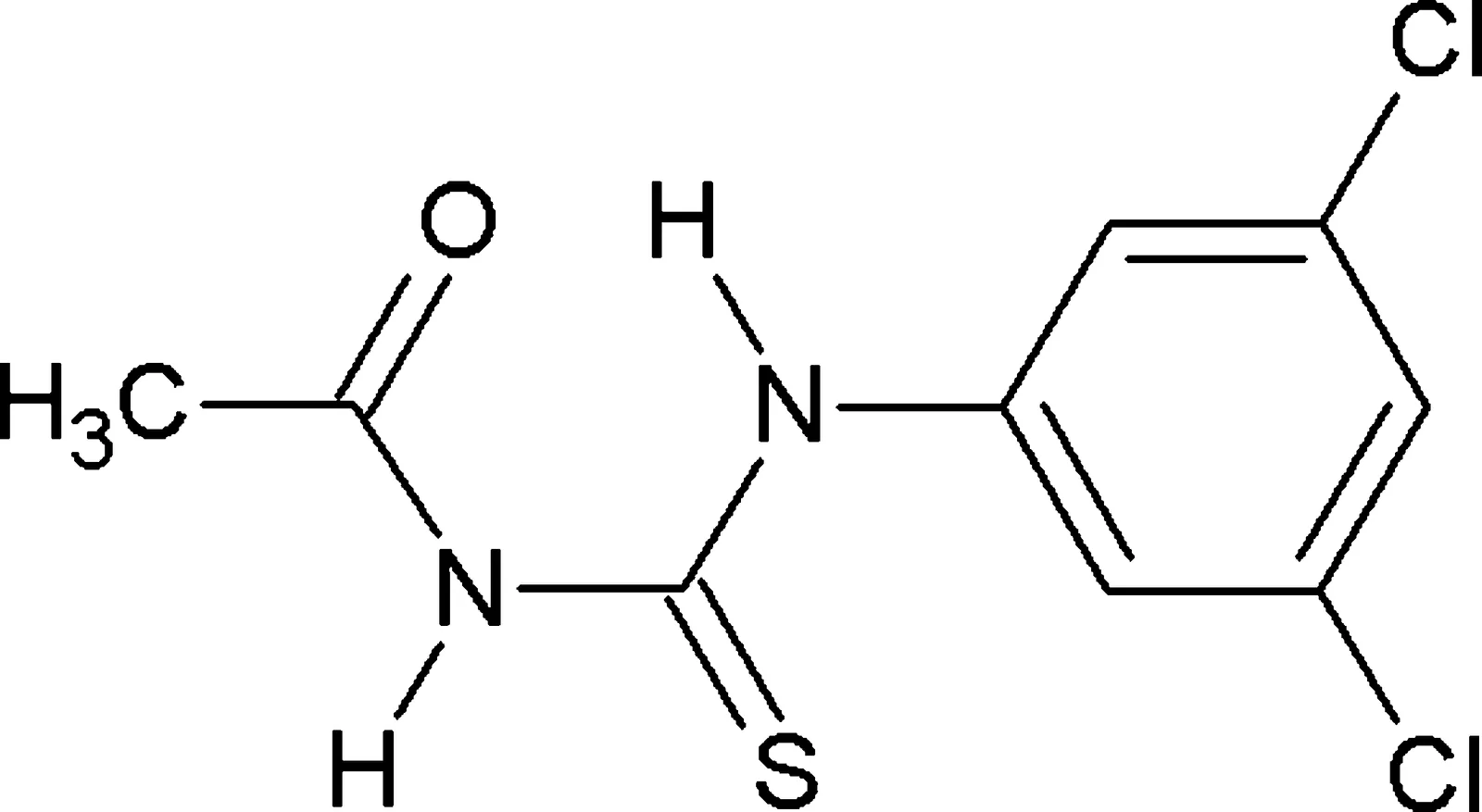


## 2. Structural commentary

**Figure 1:**
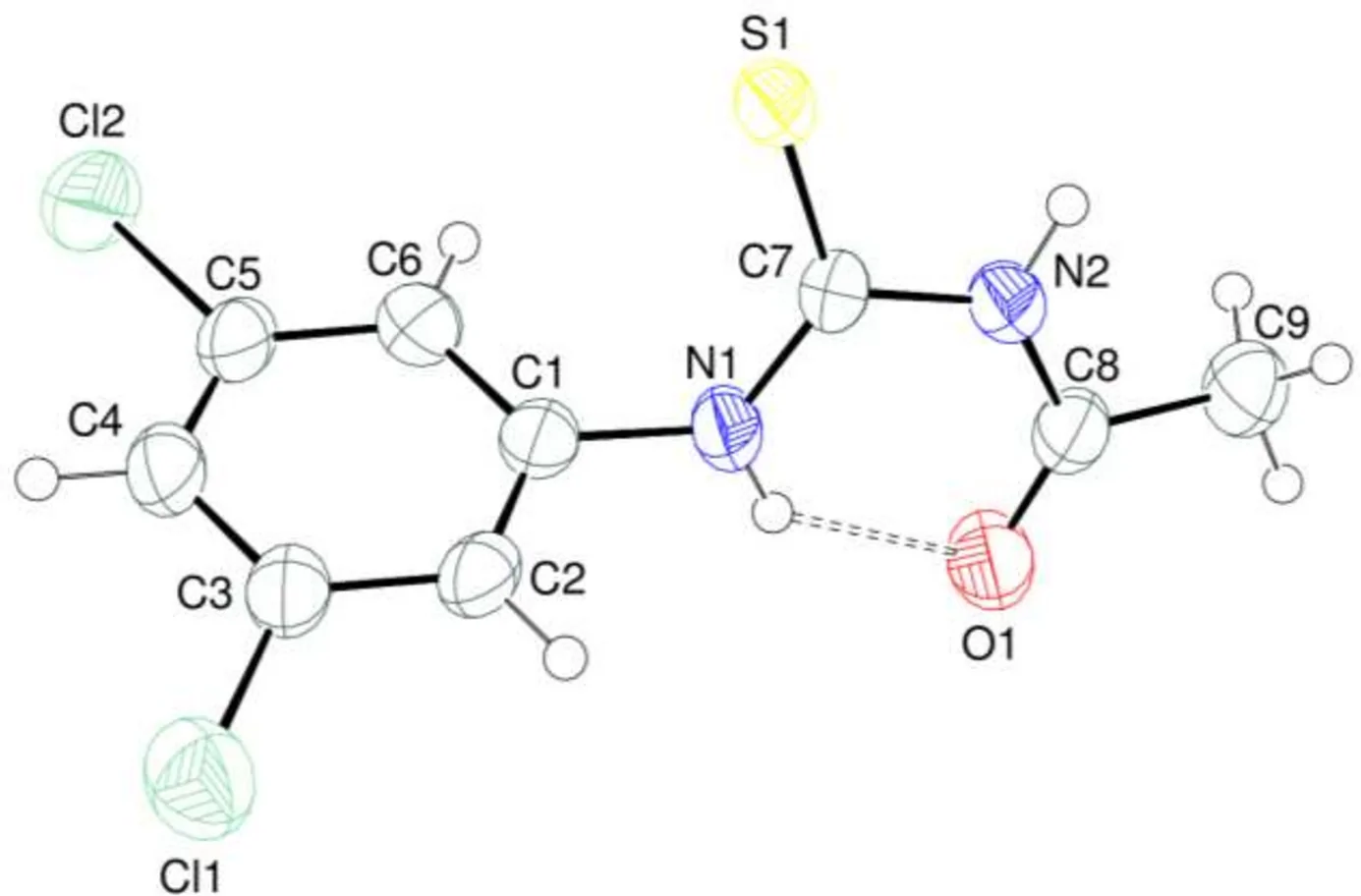
The asymmetric unit of (**I**) with displacement ellipsoids drawn at the 50% probability level. The intra­molecular N—H⋯O hydrogen bond is shown as a dashed line. Only the major components of the disordered methyl H atoms are shown for clarity.

Compound (**I**) consists of di­chloro­phenyl and acetyl moieties bridged by a thio­urea group (Fig. 1 ▸). The two N—H bonds, like the thio­carbonyl C=S and carbonyl C=O bonds, are in pseudo-*anti* conformations. The acetyl (O1/C8/C9) group is oriented with respect to the statistically planar thio­urea (S1/C7/N1/N2) group [root-mean-square value of 0.004 (4) Å] at a dihedral angle of 5.6 (6)°; the dihedral angles between the phenyl ring (C1–C6) and acetyl and thio­urea groups are 70.3 (4) and 69.1 (1)°, respectively. The Cl1, Cl2 and N1 atoms are −0.037 (2), −0.016 (2) and −0.034 (4) Å,respectively, away from the least-squares plane of the phenyl ring. All bond lengths are in normal ranges (Allen *et al.*, 1987 ▸) and comparable to those in the similar compounds *N*-(2-chloro­phen­yl)-*N*′-propanoyl-thio­urea (**II**) (Kumar & Hökelek, 2026**a* ▸)* and *N*-acetyl-*N*′-(4-chloro­phen­yl) thio­urea (**III**) (Kumar & Hökelek, 2026*b* ▸). An intra­molecular N—H⋯O hydrogen bond (Table 1 ▸) forms an S(6) ring motif (Etter *et al.*, 1990 ▸) and stabilizes the mol­ecular conformation (Fig. 1 ▸).

The C1—N1—C7 [123.9 (4)°], S1—C7—N1 [124.4 (3)°] and N2—C8—C9 [113.6 (4)°] bond angles in (**I**) are significantly narrowed when compared with the corresponding values in compounds (**II**) and (**III**). Comparison with other structures is provided in the Database survey.

## 3. Supra­molecular features

**Figure 2:**
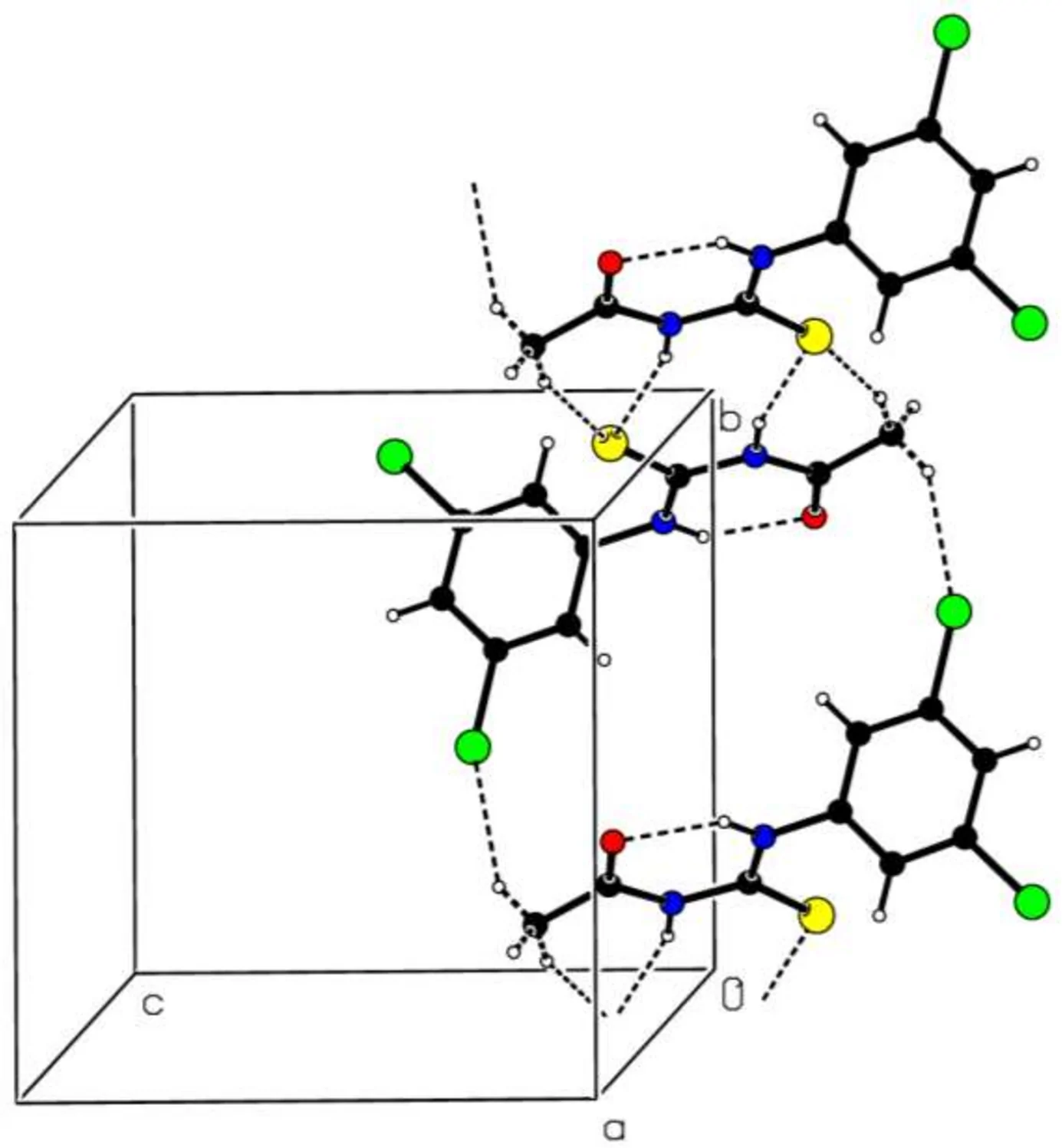
A partial packing diagram showing the infinite hydrogen-bonded chains extending parallel to the *b* axis. The intra­molecular N—H⋯O and inter­molecular N—H⋯S, C—H⋯S and C—H⋯Cl hydrogen bonds are shown as dashed lines with the *S*(6), 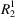
(6), 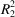
(8) and 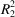
(20) ring motifs. Only the hydrogen-bonded components of the disordered atoms are shown for clarity.

In the crystal, C—H⋯Cl and double-acceptor N—H⋯S and C—H⋯S hydrogen bonds (Table 1 ▸) link the mol­ecules into infinite chains extending parallel to the *b* axis, enclosing 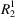
(6), 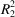
(8) and 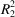
(20) ring motifs (Etter *et al.*, 1990 ▸; Fig. 2 ▸). Significant π⋯π or C—H⋯π(ring) inter­actions are not observed.

**Figure 3:**
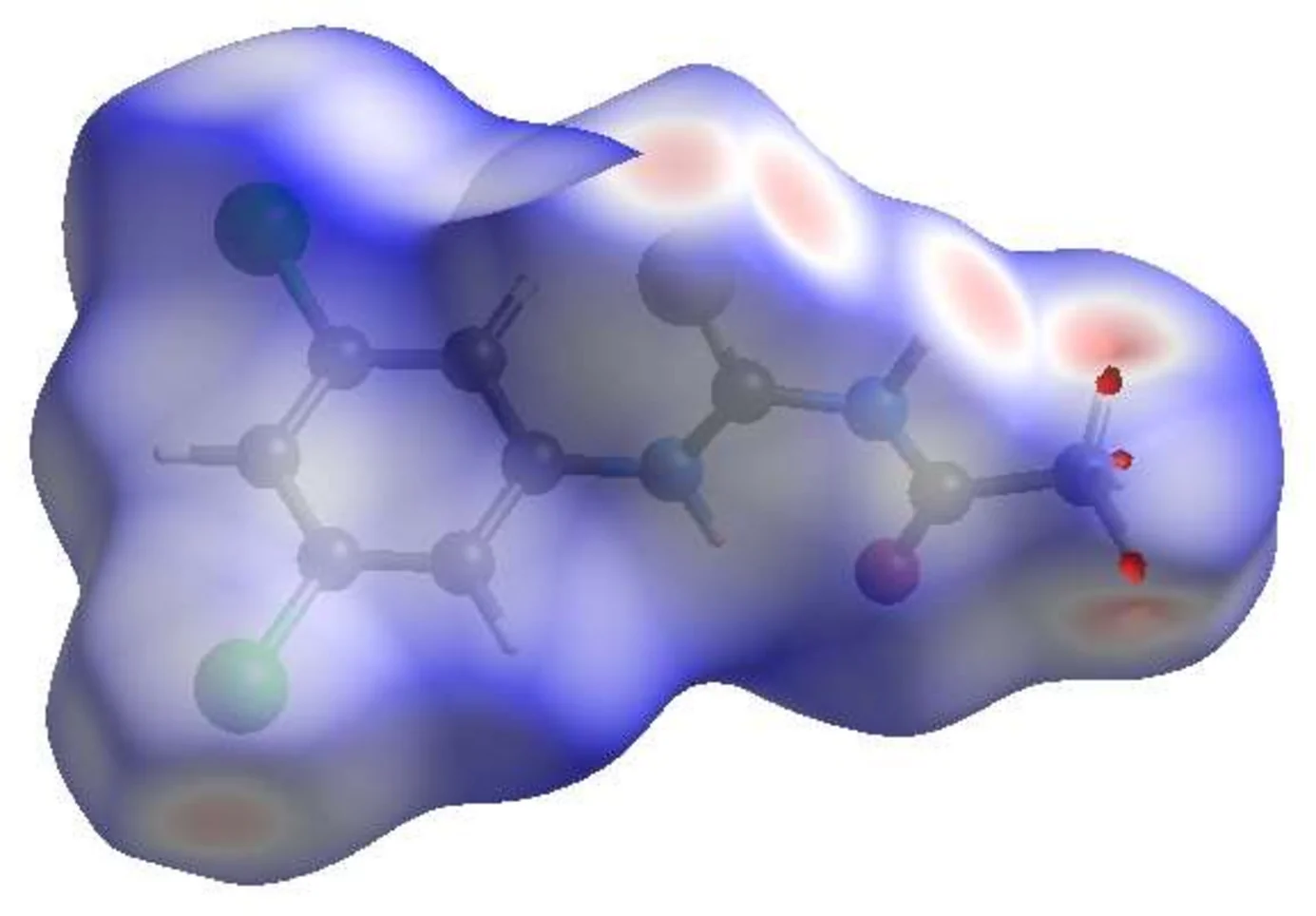
View of the three-dimensional Hirshfeld surface of (**I**) plotted over *d*_norm_.

**Figure 4:**
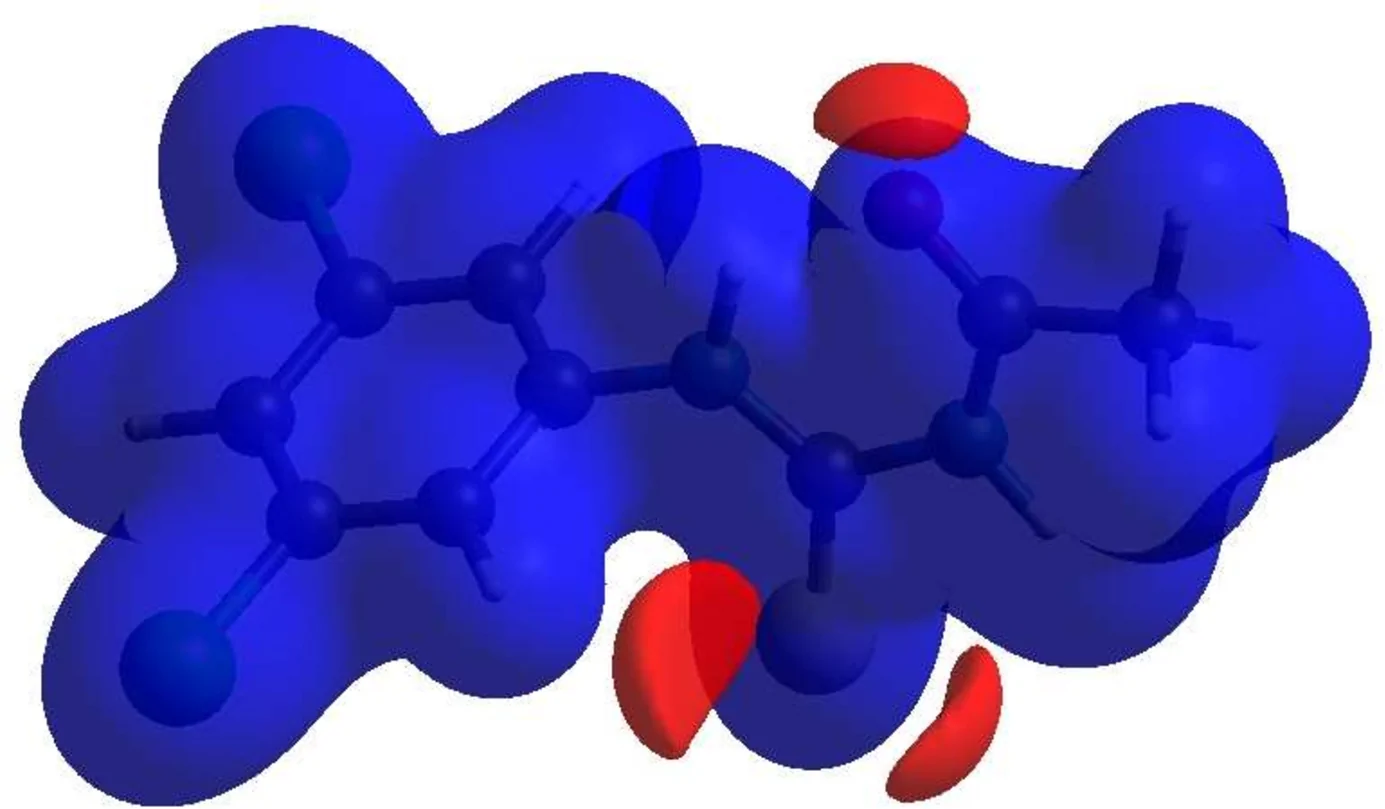
View of the three-dimensional Hirshfeld surface of the title compound plotted over electrostatic potential. Hydrogen-bonding donors and acceptors are shown as blue and red regions around the atoms, corresponding to positive and negative potentials, respectively.

The inter­molecular inter­actions in the crystal were qu­anti­fied and visualized by carrying out a Hirshfeld surface (HS) analysis using *CrystalExplorer* (Spackman *et al.*, 2021 ▸). It is noted that only the major components of the disordered part of the methyl group (C9) were taken into account for the analysis. Fig. 3 ▸ shows the HS plotted over *d*_norm_ in the crystal. The white surface indicates contacts with distances equal to the sum of van der Waals radii, and the red and blue colours indicate distances shorter (in close contact) or longer (distinct contacts) than the van der Waals radii, respectively. The red spots indicate their roles as the respective donors and/or acceptors atoms in hydrogen-bonding, as discussed. They also appear as the blue and red regions corresponding to positive and negative potentials on the HS mapped over electrostatic potential, as shown in Fig. 4 ▸. The blue and red regions indicate positive (hydrogen-bond donors) and negative (hydrogen-bond acceptors) electrostatic potentials.

**Figure 5:**
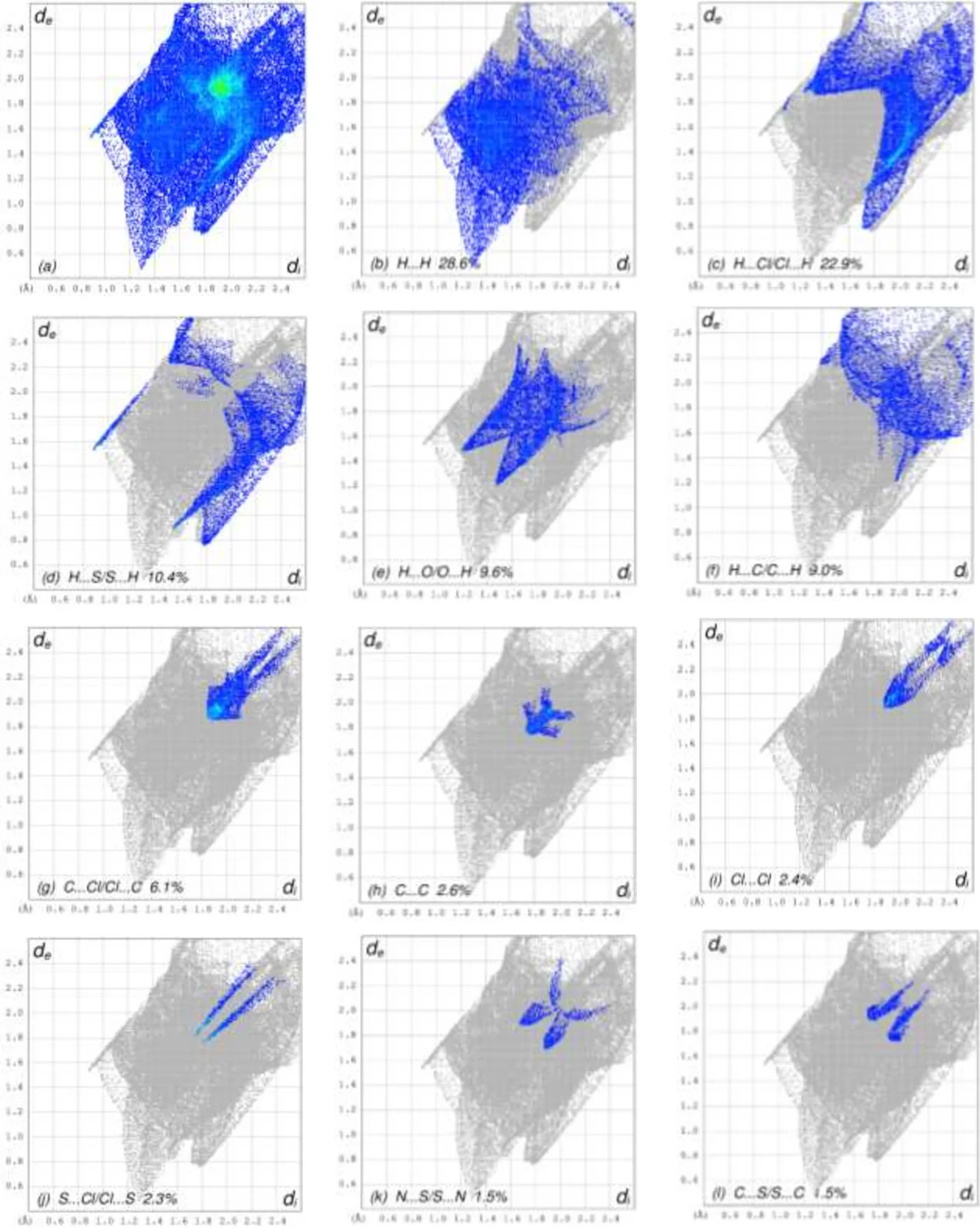
The full two-dimensional fingerprint plots for (**I**), showing (*a*) all inter­actions, and delineated into (*b*) H⋯H, (*c*) H⋯Cl/Cl⋯H, (*d*) H⋯S/S⋯H, (*e*) H⋯O/O⋯H, (*f*) H⋯C/C⋯H, (*g*) C⋯Cl/Cl⋯C, (*h*) C⋯C, (i) Cl⋯Cl, (*j*) S⋯Cl/Cl⋯S, (*k*) N⋯S/S⋯N, (*l*) C⋯S/S⋯C, (*m*) C⋯O/O⋯C, (*n*) H⋯N/N⋯H, (*o*) N⋯O/O⋯N, (*p*) O⋯Cl/Cl⋯O and (*r*) O⋯S/S⋯O inter­actions. The *d*_i_ and *d*_e_ values are the closest inter­nal and external distances (in Å) from given points on the Hirshfeld surface.

The overall two-dimensional fingerprint plot is shown in Fig. 5 ▸*a* and delineated int odifferent contact types in Fig. 5 ▸*b*–*r*. According to the two-dimensional fingerprint plots, the H⋯H, H⋯Cl/Cl⋯H, H⋯S/S⋯H, H⋯O/O⋯H and H⋯C/C⋯H contacts make the most significant contributions to the HS, at 28.6%, 22.9%, 10.4%, 9.6% and 9.0%, respectively.

**Figure 6:**
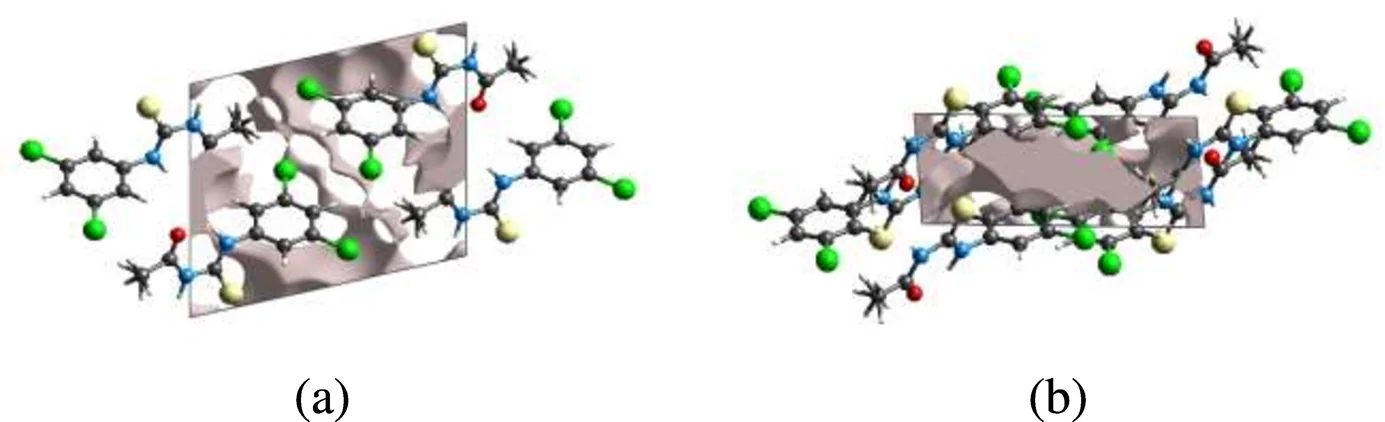
Crystal voids in the packing of (**I**) viewed down the (*a*) *a* axis and (*b*) *b* axis.

The volume of the crystal voids (Fig. 6 ▸*a*,*b*) and the percentage of free space in the unit cell was calculated as 62.70 Å^3^ and 10.86%, respectively, showing that there are no large cavities in the crystal packing.

The inter­molecular inter­action energies were computed on basis of the CE–B3LYP/6–31G(d,p) energy model available in *CrystalExplorer* (Spackman *et al.*, 2021 ▸), where a cluster of mol­ecules is generated by applying crystallographic symmetry operations with respect to a selected central mol­ecule within the radius of 3.8 Å by default. The total inter­molecular energy (*E*_tot_) is the sum of electrostatic (*E*_ele_), polarization (*E*_pol_), dispersion (*E*_dis_) and exchange-repulsion (*E*_rep_) energies (Turner *et al.*, 2015 ▸) with scale factors of 1.057, 0.740, 0.871 and 0.618, respectively (Mackenzie *et al.*, 2017 ▸).

Hydrogen bonding inter­action energies (in kJ mol^−1^) were calculated to be −7.9 (*E*_ele_), −0.9 (*E*_pol_), −16.2 (*E*_dis_), 21.2 (*E*_rep_) and −10.0 (*E*_tot_) for the fragment N2—H2N⋯S1; −1.8 (*E*_ele_), −0.2 (*E*_pol_), −3.8 (*E*_dis_), 2.5 (*E*_rep_) and −3.9 (*E*_tot_) for the fragment C9—H9F⋯S1; −2.6 (*E*_ele_), −1.0 (*E*_pol_), −6.1 (*E*_dis_), 1.6 (*E*_rep_) and −7.7 (E_tot_) for the fragment C9—H9E⋯Cl1.

**Figure 7:**
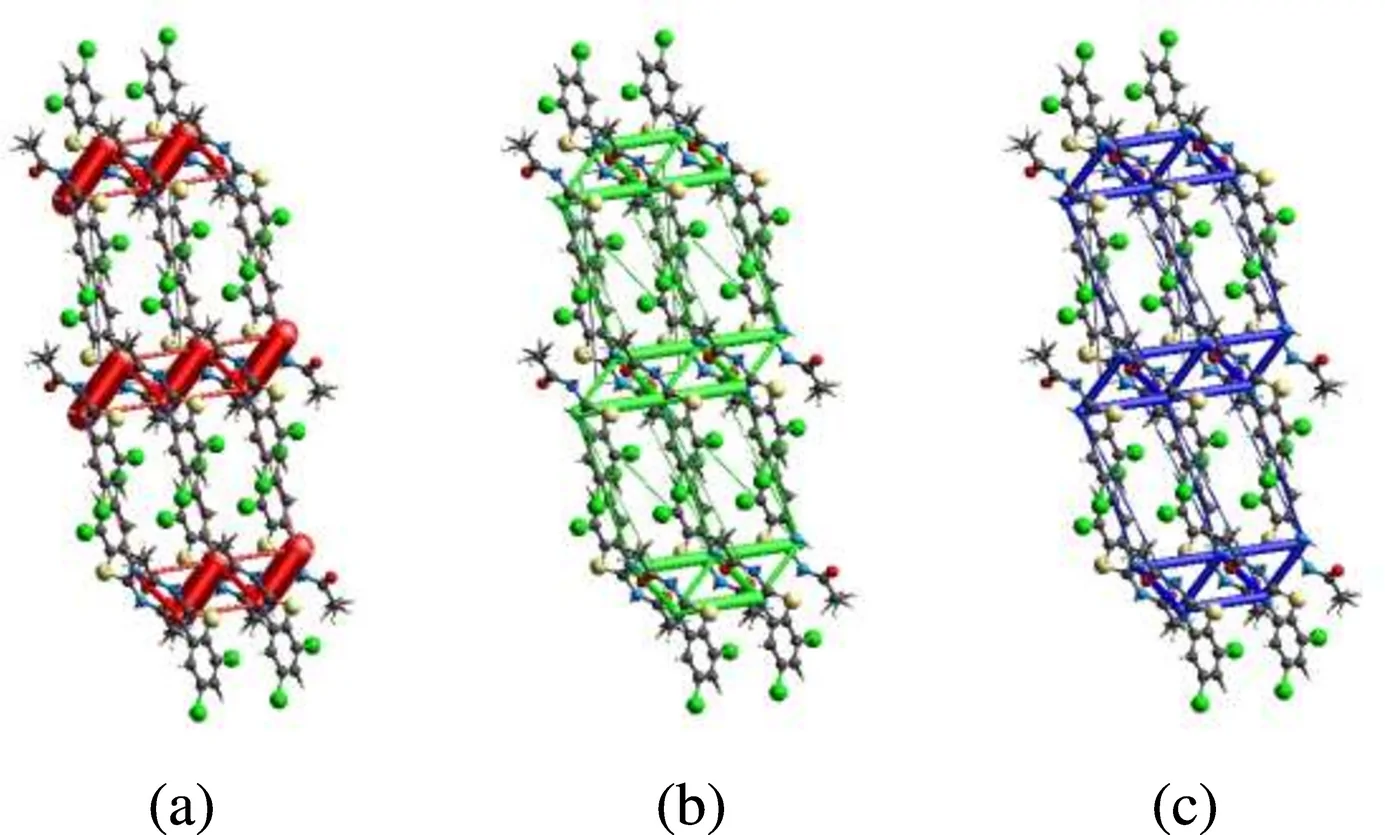
The energy frameworks for a cluster of mol­ecules viewed down the *b* axis showing the (*a*) electrostatic energy, (*b*) dispersion energy and (*c*) total energy diagrams. The cylindrical radius is proportional to the relative strength of the corresponding energies and they were adjusted to the same scale factor of 80 with cut-off value of 5 kJ mol^−1^ within 2×2×2 unit cells.

Energy frameworks combine the calculation of inter­molecular inter­action energies with a graphical representation of their magnitudes, in which they were constructed for *E*_ele_ (red cylinders), *E*_dis_ (green cylinders) and *E*_tot_ (blue cylinders) (Fig. 7 ▸*a*–*c*). The evaluation of the electrostatic, dispersion and total energy frameworks indicates that the stabilization in the crystal structure is dominated *via* the dispersion energy contributions.

## 4. Database survey

A search of the Cambridge Structural Database (CSD, Version 6.00, updated May 2025; Groom *et al.*, 2016 ▸) identified eleven structurally related structures of *N*-acetyl-*N*′-aryl­thio­urea derivatives. These include: 3-acetyl-1-phenyl­thio­urea, C_9_H_10_N_2_OS (CSD refcode GARBIM; Shahwar *et al.*, 2012*a* ▸), 3-acetyl-1-(2-methyl­phen­yl)thio­urea, C_10_H_12_N_2_OS (YAXNUI; Shahwar *et al.*, 2012*b* ▸), 3-acetyl-1-(3-chloro­phen­yl)thio­urea, C_9_H_9_ClN_2_OS (YAXTIC; Shahwar *et al.*, 2012*c* ▸), 3-acetyl-1-(3-methyl­phen­yl)thio­urea, C_10_H_12_N_2_OS (LEGQEV; Gowda *et al.*, 2012*a* ▸), 3-acetyl-1-(2,5-di­methyl­phen­yl)thio­urea, C_11_H_14_N_2_OS (LEDYAW; Gowda *et al.*, 2012*b* ▸), 3-acetyl-1-(2,3-di­chloro­phen­yl)thio­urea, C_9_H_8_Cl_2_N_2_OS (LEFBEF; Gowda *et al.*, 2012*c* ▸), 3-acetyl-1-(4-methyl­phen­yl)thio­urea, C_10_H_12_N_2_OS (XAZYOO; Gowda *et al.*, 2012*d* ▸), 3-acetyl-1-(2,4-di­methyl­phen­yl)thio­urea, C_11_H_14_N_2_OS (LEGCIL; Gowda *et al.*, 2012*e* ▸), 3-acetyl-1-(2,3-di­methyl­phen­yl)thio­urea, C_11_H_14_N_2_OS (XEBKUM; Kumar *et al.*, 2012*a* ▸), 3-acetyl-1-(2,6-di­methyl­phen­yl)thio­urea, C_11_H_14_N_2_OS (LEDSOE; Kumar *et al.*, 2012*b* ▸), 3-acetyl-1-(2,6-di­chloro­phen­yl)thio­urea, C_9_H_8_Cl_2_N_2_OS (LEDSUK; Kumar *et al.*, 2012*c* ▸). Overall, these *N*-acetyl-*N*′-aryl­thio­urea derivatives all exhibit hydrogen-bonding networks dominated by N—H⋯S and N—H⋯O inter­actions, which frequently generate graph-set motifs such as 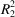
(8) and 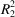
(12), and assemble the mol­ecules into one-, two- or three-dimensional supra­molecular networks. In several structures, three-center N—H⋯(O,S) hydrogen bonds and weaker C—H⋯S contacts further reinforce the crystal packing, highlighting the key role of hydrogen-bonding in consolidating this family of compounds.

## 5. Synthesis and crystallization

Compound (**I**) was prepared by adding acetyl chloride (0.10 mol) dissolved in acetone (30 ml) dropwise to a suspension of potassium thio­cyanate (0.10 mol) in acetone (30 ml). The resulting mixture was stirred for 2 h and subsequently refluxed for 30 min. After cooling to room temperature, a solution of 3,5-di­chloro­aniline (0.10 mol) in acetone (10 ml) was added, and the reaction mixture was refluxed for 3 h. The progress of the reaction mixture was monitored by thin-layer chromatography (TLC). Upon completion, the reaction mixture was poured into acidified cold water, resulting in the precipitation of the product. The solid material was collected by suction filtration, thoroughly washed with water, and dried. Colourless crystals suitable for X-ray analysis were obtained by slow evaporation of an aceto­nitrile solution. Colourless solid, yield 80%, mp. 437 K. IR (KBr) cm^−1^: 3183 (N—H str.), 1160 (C=S str.) and 1694 (C=O str.).

## 6. Refinement

Crystal data, data collection and structure refinement details are summarized in Table 2 ▸. The hydrogen atoms of the methyl group were modelled over two sets of sites, with an occupancy ratio of 0.92 (6):0.08 (6). The NH hydrogen atoms were located from a difference Fourier map and refined with a distance restraint of 0.85 (2) Å. The C-bonded hydrogen atom positions were calculated geometrically at distances of 0.93 Å (for aromatic CH) and 0.96 Å (for methyl CH) and refined using a riding model by applying the constraint *U*_iso_(H) = *k* × *U*_eq_(C, N), where *k* = 1.5 for methyl H and *k* = 1.2 for the other H atoms.

## Supplementary Material

Crystal structure: contains datablock(s) I, global. DOI: 10.1107/S2056989026010121/wm5812sup1.cif

Structure factors: contains datablock(s) I. DOI: 10.1107/S2056989026010121/wm5812Isup2.hkl

Supporting information file. DOI: 10.1107/S2056989026010121/wm5812Isup3.cml

CCDC reference: 1816809

Additional supporting information:  crystallographic information; 3D view; checkCIF report

## Figures and Tables

**Table 1:** Hydrogen-bond geometry (Å, °)

*D*—H⋯*A*	*D*—H	H⋯*A*	*D*⋯*A*	*D*—H⋯*A*
N1—H1*N*⋯O1	0.86 (2)	1.95 (4)	2.655 (5)	138 (5)
N2—H2*N*⋯S1^i^	0.86 (2)	2.57 (2)	3.395 (4)	163 (5)
C9—H9*F*⋯S1^i^	0.96	2.66	3.576 (5)	160
C9—H9*E*⋯Cl1^ii^	0.96	2.62	3.478 (6)	149

Symmetry codes: (i) 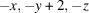; (ii) 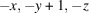.

**Table 2:** Experimental details

Crystal data	
Chemical formula	C_9_H_8_Cl_2_N_2_OS
*M*_r_	263.13
Crystal system, space group	Triclinic, *P* 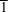
Temperature (K)	293
*a*, *b*, *c* (Å)	4.5764 (9), 10.873 (2), 12.093 (2)
α, β, γ (°)	102.87 (1), 92.65 (1), 99.04 (1)
*V* (Å^3^)	577.27 (18)
*Z*	2
Radiation type	Mo *K*α
μ (mm^−1^)	0.72
Crystal size (mm)	0.48 × 0.14 × 0.10

Data collection	
Diffractometer	Oxford Diffraction Xcalibur with Sapphire CCD
Absorption correction	Multi-scan (*CrysAlis RED*; Oxford Diffraction, 2009 ▸)
*T*_min_, *T*_max_	0.725, 0.932
No. of measured, independent and observed [*I* > 2σ(*I*)] reflections	3301, 2007, 1747
*R*_int_	0.013
(sin θ/λ)_max_ (Å^−1^)	0.599

Refinement	
*R*[*F*^2^ > 2σ(*F*^2^)], *wR*(*F*^2^), *S*	0.065, 0.139, 1.33
No. of reflections	2007
No. of parameters	144
No. of restraints	2
H-atom treatment	H atoms treated by a mixture of independent and constrained refinement
Δρ_max_, Δρ_min_ (e Å^−3^)	0.46, −0.40

Computer programs: *CrysAlis CCD* (Oxford Diffraction, 2009 ▸), *CrysAlis RED* (Oxford Diffraction, 2009 ▸), *SHELXT* (Sheldrick, 2015*a* ▸), *SHELXL* (Sheldrick, 2015*b* ▸), *ORTEP-3* for Windows (Farrugia, 2012 ▸), *WinGX* (Farrugia, 2012 ▸) and *PLATON* (Spek, 2009 ▸).

## supplementary crystallographic information

### 1-Acetyl-3-(3,5-dichlorophenyl)thiourea Crystal data

**Table:**

C_9_H_8_Cl_2_N_2_OS	*Z* = 2
*M**_r_* = 263.13	*F*(000) = 268
Triclinic, *P*1	*D*_x_ = 1.514 Mg m^−^^3^
*a* = 4.5764 (9) Å	Mo *K*α radiation, λ = 0.71073 Å
*b* = 10.873 (2) Å	Cell parameters from 1807 reflections
*c* = 12.093 (2) Å	θ = 2.9–27.6°
α = 102.87 (1)°	µ = 0.72 mm^−^^1^
β = 92.65 (1)°	*T* = 293 K
γ = 99.04 (1)°	Needle, colourless
*V* = 577.27 (18) Å^3^	0.48 × 0.14 × 0.10 mm

### 1-Acetyl-3-(3,5-dichlorophenyl)thiourea Data collection

**Table:**

Oxford Diffraction Xcalibur with Sapphire CCD diffractometer	1747 reflections with *I* > 2σ(*I*)
Rotation method data acquisition using ω scans.	*R*_int_ = 0.013
Absorption correction: multi-scan (*CrysAlisRED*; Oxford Diffraction, 2009)	θ_max_ = 25.2°, θ_min_ = 2.9°
*T*_min_ = 0.725, *T*_max_ = 0.932	*h* = −5→5
3301 measured reflections	*k* = −12→13
2007 independent reflections	*l* = −14→14

### 1-Acetyl-3-(3,5-dichlorophenyl)thiourea Refinement

**Table:**

Refinement on *F*^2^	2 restraints
Least-squares matrix: full	Hydrogen site location: mixed
*R*[*F*^2^ > 2σ(*F*^2^)] = 0.065	H atoms treated by a mixture of independent and constrained refinement
*wR*(*F*^2^) = 0.139	*w* = 1/[σ^2^(*F*_o_^2^) + (0.0189*P*)^2^ + 1.3739*P*] where *P* = (*F*_o_^2^ + 2*F*_c_^2^)/3
*S* = 1.33	(Δ/σ)_max_ < 0.001
2007 reflections	Δρ_max_ = 0.46 e Å^−^^3^
144 parameters	Δρ_min_ = −0.40 e Å^−^^3^

### 1-Acetyl-3-(3,5-dichlorophenyl)thiourea Special details

**Table:**

Geometry. All esds (except the esd in the dihedral angle between two l.s. planes) are estimated using the full covariance matrix. The cell esds are taken into account individually in the estimation of esds in distances, angles and torsion angles; correlations between esds in cell parameters are only used when they are defined by crystal symmetry. An approximate (isotropic) treatment of cell esds is used for estimating esds involving l.s. planes.

### 1-Acetyl-3-(3,5-dichlorophenyl)thiourea Fractional atomic coordinates and isotropic or equivalent isotropic displacement parameters (Å^2^)

**Table:**

	*x*	*y*	*z*	*U*_iso_*/*U*_eq_	Occ. (<1)
C1	−0.1146 (11)	0.7042 (4)	0.2407 (4)	0.0450 (11)	
C2	0.0424 (11)	0.6056 (4)	0.2412 (4)	0.0450 (11)	
H2	0.074750	0.551893	0.173074	0.054*	
C3	0.1511 (10)	0.5877 (5)	0.3442 (4)	0.0463 (11)	
C4	0.1073 (12)	0.6672 (5)	0.4456 (4)	0.0531 (13)	
H4	0.183289	0.655586	0.514480	0.064*	
C5	−0.0512 (13)	0.7642 (5)	0.4430 (4)	0.0544 (14)	
C6	−0.1656 (12)	0.7838 (5)	0.3414 (4)	0.0518 (13)	
H6	−0.274366	0.849115	0.341043	0.062*	
C7	−0.1459 (10)	0.8188 (4)	0.0902 (3)	0.0380 (10)	
C8	−0.5341 (11)	0.7346 (5)	−0.0725 (4)	0.0477 (12)	
C9	−0.6596 (12)	0.7745 (5)	−0.1735 (4)	0.0566 (13)	
H9A	−0.504337	0.791366	−0.221559	0.085*	0.92 (6)
H9B	−0.743052	0.850490	−0.147804	0.085*	0.92 (6)
H9C	−0.811532	0.706967	−0.215545	0.085*	0.92 (6)
H9D	−0.868277	0.774516	−0.168380	0.085*	0.08 (6)
H9E	−0.629562	0.715392	−0.242135	0.085*	0.08 (6)
H9F	−0.561082	0.858915	−0.174394	0.085*	0.08 (6)
N1	−0.2353 (10)	0.7195 (4)	0.1336 (3)	0.0484 (10)	
N2	−0.2930 (9)	0.8207 (4)	−0.0129 (3)	0.0424 (9)	
O1	−0.6361 (9)	0.6373 (4)	−0.0442 (3)	0.0731 (12)	
Cl1	0.3434 (3)	0.46234 (15)	0.34501 (13)	0.0684 (4)	
Cl2	−0.1133 (5)	0.86361 (15)	0.57162 (12)	0.0870 (6)	
S1	0.1331 (3)	0.93861 (12)	0.14923 (10)	0.0476 (3)	
H1N	−0.376 (8)	0.662 (4)	0.095 (4)	0.057*	
H2N	−0.231 (11)	0.890 (3)	−0.033 (4)	0.057*	

### 1-Acetyl-3-(3,5-dichlorophenyl)thiourea Atomic displacement parameters (Å^2^)

**Table:**

	*U*^11^	*U*^22^	*U*^33^	*U*^12^	*U*^13^	*U*^23^
C1	0.054 (3)	0.039 (3)	0.036 (2)	−0.011 (2)	0.002 (2)	0.010 (2)
C2	0.052 (3)	0.042 (3)	0.033 (2)	−0.009 (2)	0.005 (2)	0.005 (2)
C3	0.047 (3)	0.048 (3)	0.040 (3)	−0.009 (2)	−0.002 (2)	0.013 (2)
C4	0.065 (3)	0.050 (3)	0.039 (3)	−0.012 (3)	−0.006 (2)	0.018 (2)
C5	0.082 (4)	0.045 (3)	0.029 (2)	−0.011 (3)	0.010 (2)	0.008 (2)
C6	0.069 (3)	0.042 (3)	0.043 (3)	0.001 (2)	0.007 (2)	0.012 (2)
C7	0.045 (3)	0.041 (2)	0.027 (2)	0.007 (2)	0.0044 (18)	0.0066 (18)
C8	0.054 (3)	0.049 (3)	0.035 (2)	−0.002 (2)	0.001 (2)	0.006 (2)
C9	0.057 (3)	0.062 (3)	0.046 (3)	0.001 (3)	−0.009 (2)	0.013 (3)
N1	0.062 (3)	0.045 (2)	0.034 (2)	−0.0096 (19)	−0.0047 (18)	0.0165 (18)
N2	0.047 (2)	0.041 (2)	0.038 (2)	0.0003 (18)	0.0022 (17)	0.0138 (17)
O1	0.089 (3)	0.065 (3)	0.053 (2)	−0.029 (2)	−0.017 (2)	0.0222 (19)
Cl1	0.0651 (9)	0.0730 (10)	0.0703 (9)	0.0123 (7)	−0.0036 (7)	0.0250 (8)
Cl2	0.1534 (17)	0.0627 (9)	0.0380 (7)	0.0055 (10)	0.0212 (9)	0.0037 (6)
S1	0.0534 (7)	0.0452 (7)	0.0416 (7)	−0.0070 (5)	−0.0059 (5)	0.0173 (5)

### 1-Acetyl-3-(3,5-dichlorophenyl)thiourea Geometric parameters (Å, º)

**Table:**

C1—C6	1.381 (7)	C7—N2	1.396 (5)
C1—C2	1.382 (7)	C7—S1	1.678 (4)
C1—N1	1.438 (6)	C8—O1	1.216 (6)
C2—C3	1.386 (6)	C8—N2	1.384 (6)
C2—H2	0.9300	C8—C9	1.502 (7)
C3—C4	1.377 (7)	C9—H9A	0.9600
C3—Cl1	1.736 (5)	C9—H9B	0.9600
C4—C5	1.376 (7)	C9—H9C	0.9600
C4—H4	0.9300	C9—H9D	0.9600
C5—C6	1.386 (7)	C9—H9E	0.9600
C5—Cl2	1.749 (5)	C9—H9F	0.9600
C6—H6	0.9300	N1—H1N	0.859 (19)
C7—N1	1.320 (6)	N2—H2N	0.858 (19)
			
C6—C1—C2	120.8 (5)	H9A—C9—H9B	109.5
C6—C1—N1	120.3 (5)	C8—C9—H9C	109.5
C2—C1—N1	118.9 (4)	H9A—C9—H9C	109.5
C1—C2—C3	119.2 (4)	H9B—C9—H9C	109.5
C1—C2—H2	120.4	C8—C9—H9D	109.5
C3—C2—H2	120.4	H9A—C9—H9D	141.1
C4—C3—C2	121.0 (5)	H9B—C9—H9D	56.3
C4—C3—Cl1	119.8 (4)	H9C—C9—H9D	56.3
C2—C3—Cl1	119.3 (4)	C8—C9—H9E	109.5
C5—C4—C3	118.7 (5)	H9A—C9—H9E	56.3
C5—C4—H4	120.7	H9B—C9—H9E	141.1
C3—C4—H4	120.7	H9C—C9—H9E	56.3
C4—C5—C6	121.7 (5)	H9D—C9—H9E	109.5
C4—C5—Cl2	118.8 (4)	C8—C9—H9F	109.5
C6—C5—Cl2	119.4 (4)	H9A—C9—H9F	56.3
C1—C6—C5	118.6 (5)	H9B—C9—H9F	56.3
C1—C6—H6	120.7	H9C—C9—H9F	141.1
C5—C6—H6	120.7	H9D—C9—H9F	109.5
N1—C7—N2	116.6 (4)	H9E—C9—H9F	109.5
N1—C7—S1	124.4 (3)	C7—N1—C1	123.9 (4)
N2—C7—S1	119.0 (3)	C7—N1—H1N	116 (4)
O1—C8—N2	122.8 (4)	C1—N1—H1N	120 (4)
O1—C8—C9	123.6 (4)	C8—N2—C7	127.8 (4)
N2—C8—C9	113.6 (4)	C8—N2—H2N	121 (4)
C8—C9—H9A	109.5	C7—N2—H2N	111 (4)
C8—C9—H9B	109.5		
			
C6—C1—C2—C3	−0.5 (7)	C4—C5—C6—C1	−0.8 (8)
N1—C1—C2—C3	−177.9 (4)	Cl2—C5—C6—C1	−179.9 (4)
C1—C2—C3—C4	−0.6 (7)	N2—C7—N1—C1	−178.1 (4)
C1—C2—C3—Cl1	179.0 (3)	S1—C7—N1—C1	3.3 (7)
C2—C3—C4—C5	1.0 (7)	C6—C1—N1—C7	68.6 (7)
Cl1—C3—C4—C5	−178.6 (4)	C2—C1—N1—C7	−113.9 (5)
C3—C4—C5—C6	−0.3 (8)	O1—C8—N2—C7	−5.9 (8)
C3—C4—C5—Cl2	178.8 (4)	C9—C8—N2—C7	173.0 (4)
C2—C1—C6—C5	1.2 (7)	N1—C7—N2—C8	4.2 (7)
N1—C1—C6—C5	178.6 (4)	S1—C7—N2—C8	−177.1 (4)

### 1-Acetyl-3-(3,5-dichlorophenyl)thiourea Hydrogen-bond geometry (Å, º)

**Table:**

*D*—H···*A*	*D*—H	H···*A*	*D*···*A*	*D*—H···*A*
N1—H1*N*···O1	0.86 (2)	1.95 (4)	2.655 (5)	138 (5)
N2—H2*N*···S1^i^	0.86 (2)	2.57 (2)	3.395 (4)	163 (5)
C9—H9*F*···S1^i^	0.96	2.66	3.576 (5)	160
C9—H9*E*···Cl1^ii^	0.96	2.62	3.478 (6)	149

Symmetry codes: (i) −*x*, −*y*+2, −*z*; (ii) −*x*, −*y*+1, −*z*.
